# Temporal association between adjunctive bicyclol initiation and discordant biochemical trends in severe early-onset pregnancy-associated cholestasis: a Case Report

**DOI:** 10.3389/fphar.2026.1874712

**Published:** 2026-08-21

**Authors:** Chun-Fei Wang, Qiang Wei, Kui Yao

**Affiliations:** 1 Department of Obstetrics and Gynecology, West China Second University Hospital, Sichuan University, Chengdu, China; 2 Key Laboratory of Birth Defects and Related Diseases of Women and Children, Sichuan University, Ministry of Education, Chengdu, China

**Keywords:** alanine aminotransferase, aspartate aminotransferase, bicyclol, pregnancy-associated cholestasis, total bile acid, ursodeoxycholic acid

## Abstract

**Background:**

Severe early-onset pregnancy-associated cholestasis with atypical features may reflect an intrahepatic cholestasis of pregnancy (ICP) component superimposed on underlying hepatobiliary disease. Evidence for bicyclol use during pregnancy is very limited.

**Case presentation:**

A 34-year-old pregnant woman, developed palmoplantar pruritus and severe cholestasis at 15 weeks of gestation. She had previously undergone cholecystectomy and common bile duct exploration. The working diagnosis was pregnancy-associated cholestasis with a possible ICP component superimposed on suspected chronic hepatobiliary dysfunction. Biochemical abnormalities persisted despite treatment with ursodeoxycholic acid, ademetionine, glutathione, and polyene phosphatidylcholine. Off-label bicyclol at 50 mg three times daily was added at 29 weeks and continued for approximately 5 weeks. Before bicyclol was added, alanine aminotransferase (ALT) level ranged from 724 to 1,492 U/L, and total bile acids (TBA) level ranged from 111.2 to 224.6 μmol/L. Following bicyclol initiation, ALT ranged from 137 to 464 U/L. TBA remained markedly elevated, ranging from 96.7 to 146.6 μmol/L. Total bilirubin ranged from 42.8 to 46.5 μmol/L. Overall, aminotransferase levels were lower than the pretreatment range, whereas TBA and total bilirubin remained markedly elevated. Cesarean delivery was performed at 34+1 weeks of gestation because of worsening maternal symptoms and persistent hyperbilirubinemia. Grade III meconium-stained amniotic fluid was noted during surgery. A liveborn female infant was delivered with Apgar scores of 10, 10, and 10 at 1, 5, and 10 min, respectively. No early neonatal complications were observed. At 42 days postpartum, liver tests remained mildly abnormal, which suggested persistent hepatobiliary dysfunction rather than cholestasis limited to pregnancy.

**Conclusion:**

A temporal association was observed between bicyclol initiation and lower aminotransferase levels. However, this temporal association should not be interpreted as evidence of causality because multiple factors, including substantial pretreatment biochemical variability, concomitant multidrug therapy, advancing gestation, and possible underlying hepatobiliary disease, may have contributed to the observed changes. This case does not establish efficacy, pregnancy prolongation, or maternal and fetal safety. Instead, it provides a hypothesis-generating observation and highlights the need for further structural and genetic evaluation and systematic studies with long-term infant follow-up.

## 1 Introduction

Intrahepatic cholestasis of pregnancy (ICP) is a pregnancy-specific liver disorder usually diagnosed when pruritus and elevated serum bile acids occur after other causes have been assessed. It most often develops in the late second or third trimester and resolves after delivery. ICP is associated with preterm birth, meconium-stained amniotic fluid, and stillbirth. In singleton pregnancies, the risk of stillbirth rises markedly when peak total bile acid (TBA) concentrations reach 100 μmol/L or higher (Ovadia et al., 2019; European Association for the Study of the Liver, 2023).

Ursodeoxycholic acid (UDCA) is widely used as first-line pharmacological treatment for maternal symptoms. However, its effect on major perinatal outcomes remains uncertain (Chappell et al., 2019; Ovadia et al., 2021; European Association for the Study of the Liver, 2023). Very early onset, persistent hyperbilirubinemia, bile duct dilation, or incomplete biochemical resolution after delivery are atypical for classical ICP. These findings should prompt evaluation for coexisting structural or genetic hepatobiliary disease, including disorders involving bile-transporter genes such as *ABCB4* and *ABCB11* (European Association for the Study of the Liver, 2023; 2024). Management is particularly difficult when pregnancy-related cholestasis may overlap with impaired bile drainage or chronic cholangiopathy.

Bicyclol is a synthetic hepatoprotective agent with effects on inflammation and oxidative stress. Experimental and clinical studies in nonpregnant populations suggest that bicyclol may reduce hepatocellular injury by regulating oxidative stress, inflammatory pathways, and mitochondrial function (Zhao et al., 2021). A multicenter randomized phase II trial evaluated bicyclol in nonpregnant adults with idiosyncratic drug-induced liver injury. At doses of 25 or 50 mg three times daily, bicyclol produced higher alanine aminotransferase (ALT) normalization rates than polyene phosphatidylcholine (Tang et al., 2022). However, evidence for its use in pregnancy-associated cholestasis remains extremely limited and fetal safety has not been established.

Here, we describe severe cholestasis beginning at 15 weeks of gestation in a woman with previous cholecystectomy and common bile duct exploration. The working diagnosis was pregnancy-associated cholestasis, with a possible ICP component superimposed on suspected chronic hepatobiliary dysfunction. Bicyclol was introduced as an adjunct to ongoing UDCA-based combination therapy. After bicyclol initiation, the recorded ALT and TBA ranges were lower, whereas total bilirubin continued to rise. This report focuses on the temporal association between bicyclol initiation and discordant biochemical changes. It also discusses the diagnostic uncertainty and the rationale for adjunctive treatment. It does not establish treatment efficacy.

## 2 Case presentation

### 2.1 Patient history and initial presentation

A 34-year-old pregnant woman, gravida 3 para 1, with one previous biochemical pregnancy, presented at 15 weeks of gestation with pruritus involving the palms and soles. Her menstrual cycles were regular, and her last menstrual period was 29 June 2025. Before referral, ALT was 382 U/L, aspartate aminotransferase (AST) was 791 U/L, and TBA were 53.8 μmol/L. Liver magnetic resonance imaging showed mild extrahepatic duct dilatation, with a common bile duct diameter of up to 10 mm; no obstructing lesion was reported. Oral UDCA and ademetionine were started.

Her medical history was notable for laparoscopic cholecystectomy with common bile duct exploration in 2023 for cholelithiasis and choledocholithiasis. Her first pregnancy in 2015 had been labelled severe ICP, with ALT greater than 800 U/L and TBA of 70 μmol/L. She received UDCA and delivered a liveborn infant by cesarean delivery at 36 weeks. She had one biochemical pregnancy in 2022 and no recorded history of viral hepatitis, diagnosed autoimmune liver disease, or clinically significant alcohol use.

### 2.2 Diagnostic assessment and initial management

Shortly after referral at 15 weeks, ALT was 909 U/L, AST 465 U/L, TBA 224.6 μmol/L, and total bilirubin 12.9 μmol/L. She was admitted for evaluation. Serologic tests for hepatitis A, B, C, and E viruses, Epstein-Barr virus, cytomegalovirus, and reported TORCH infections were negative. Antinuclear, anti-smooth muscle, and anti-liver kidney microsomal antibodies were negative, as was the direct antiglobulin test. Transient elastography showed no advanced fibrosis. These findings made several infectious and autoimmune causes less likely but did not exclude other structural, metabolic, autoimmune, or genetic causes of cholestasis.

During hospitalization, she received UDCA 250 mg four times daily (1,000 mg/day), ademetionine 500 mg twice daily, and glutathione 400 mg three times daily.

Gastroenterologists, hepatobiliary surgeons, obstetricians, and clinical pharmacists reviewed the case. After counseling about diagnostic uncertainty, progressive maternal liver dysfunction, fetal risk, and prematurity, the patient and her family chose to continue the pregnancy with close monitoring.

### 2.3 Follow-up before bicyclol and disease progression

The patient was reviewed weekly. Polyene phosphatidylcholine 456 mg three times daily was added at 20 weeks of gestation. Before bicyclol initiation, from 15 + 3 to 28+1 weeks, ALT ranged from 724 to 1,492 U/L, AST from 337 to 888 U/L, TBA from 111.2 to 224.6 μmol/L, and total bilirubin from 12.9 to 43.5 μmol/L. These serial measurements showed marked pretreatment variability. Pruritus was documented but was not assessed using a validated scale.

Serial fetal ultrasonography showed mild enlargement of the fetal gallbladder without another structural abnormality. Non-invasive prenatal testing failed repeatedly because of a low fetal fraction, and the patient declined amniocentesis. Weekly nonstress testing and umbilical artery Doppler assessment showed no persistent evidence of fetal compromise.

### 2.4 Bicyclol administration and subsequent biochemical changes

At 29 weeks of gestation, the patient weighed 67 kg and received a daily UDCA dose of approximately 14.9 mg/kg. Because marked elevations in ALT and TBA persisted, oral bicyclol at 50 mg three times daily was added as an off-label adjunct. The dose was based on regimens used for liver injury in nonpregnant adults, including a phase II trial of drug-induced liver injury (Tang et al., 2022). No pregnancy-specific dosing criteria were available. Bicyclol treatment was continued for 5 weeks.

Before bicyclol initiation, liver biochemical markers showed marked fluctuations. From 15 + 3 to 28+4 weeks of gestation, ALT ranged from 724 to 1,492 U/L, AST from 337 to 888 U/L, TBA from 111.2 to 224.6 μmol/L, and total bilirubin from 12.9 to 43.5 μmol/L. The last documented pretreatment assessment at 28+4 weeks showed ALT 1,038 U/L, AST 623 U/L, TBA 201.6 μmol/L, and total bilirubin 40.2 μmol/L. The first assessment after bicyclol initiation, performed at 29+2 weeks, showed ALT 464 U/L, AST 668 U/L, TBA 122.2 μmol/L, and total bilirubin 43.6 μmol/L.

From 30 + 3 to 34+0 weeks, the recorded ALT values ranged from 137 to 218 U/L, remaining below the pretreatment range. AST remained elevated and fluctuated between 278 and 595 U/L. TBA ranged from 96.7 to 146.6 μmol/L and did not show a sustained downward trend. TBA reached 96.7 μmol/L at 31+4 weeks but increased to 146.6 μmol/L at 32+6 weeks. Total bilirubin remained elevated and fluctuated between 42.8 and 46.5 μmol/L, peaking at 46.5 μmol/L at 31+4 weeks. At the final antenatal assessment at 34+0 weeks, ALT was 137 U/L, AST was 278 U/L, TBA was 139.5 μmol/L, and total bilirubin was 45.9 μmol/L. Overall, the biochemical changes remained discordant throughout follow-up. Although the recorded aminotransferase values were lower than the pretreatment range, TBA remained markedly elevated and hyperbilirubinemia persisted.

The laboratory course is presented descriptively because of concomitant therapy and marked pretreatment variability. Pruritus was not assessed with a validated scale. No clinically apparent maternal adverse events were recorded. However, no structured adverse-event assessment was performed.

### 2.5 Delivery, neonatal observation, and postpartum course

At 34 weeks, scleral icterus and pruritus worsened, and total bilirubin remained elevated at 45.9 μmol/L. A nonstress test was reactive, and amniotic fluid and umbilical artery Doppler findings were reported as normal. After repeat multidisciplinary consultation, cesarean delivery was performed at 34+1 weeks because of progressive maternal symptoms and hyperbilirubinemia.

Grade III meconium-stained amniotic fluid was noted. A liveborn female infant was delivered with Apgar scores of 10, 10, and 10 at 1, 5, and 10 min, respectively. The birth weight was 2,490 g. No clinically apparent complication was recorded during the available early neonatal observation. This observation was not treated as evidence of drug safety.

**FIGURE 1:**
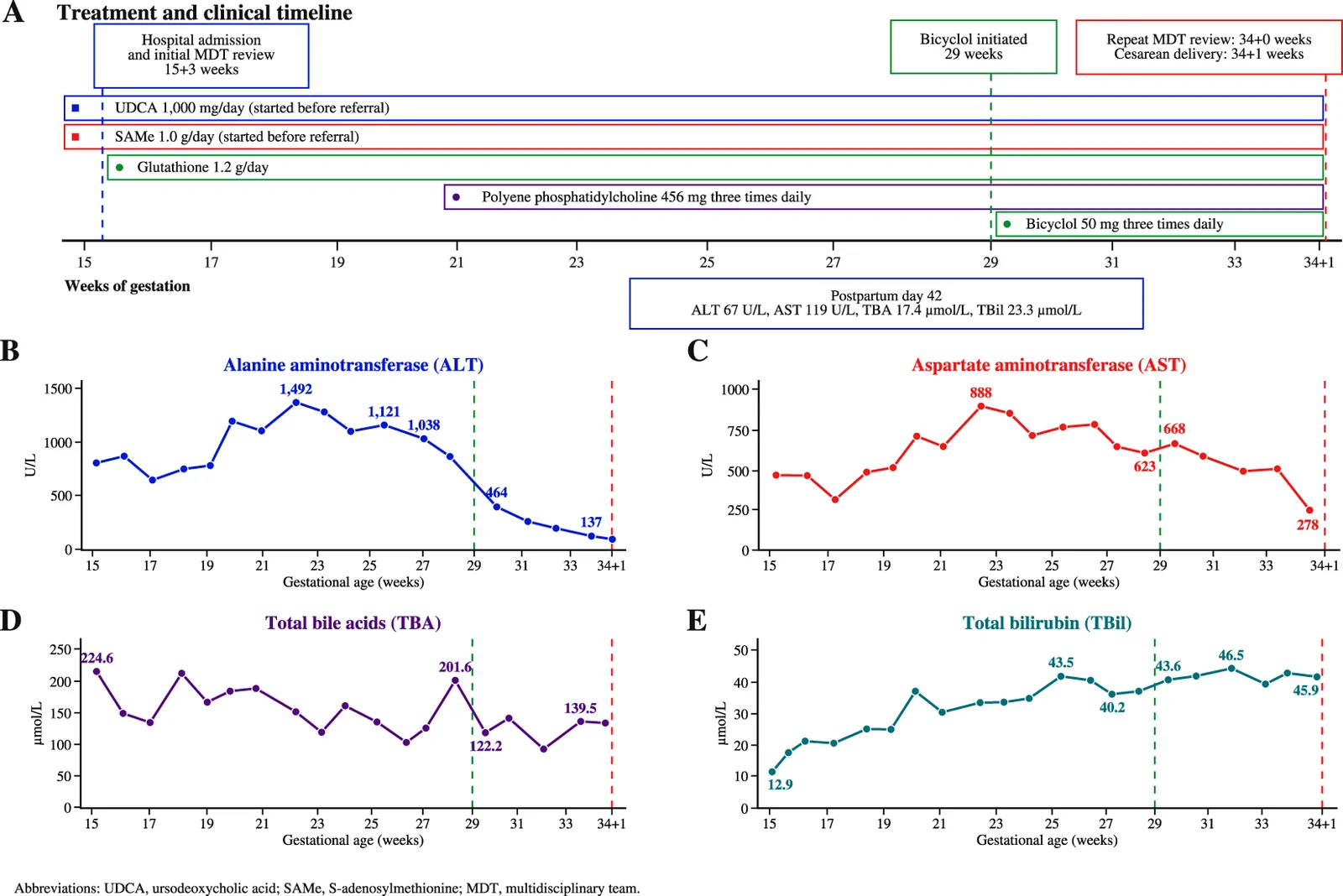
Treatment timeline and serial maternal liver biochemical changes in severe early-onset pregnancy-associated cholestasis. **(A)** Medication timelines and clinical interventions from 15 to 34 weeks gestation. **(B)** Alanine aminotransferase (ALT) levels over time. **(C)** Aspartate aminotransferase (AST) fluctuations. **(D)** Total bile acids (TBA) levels. **(E)** Total bilirubin levels.

Maternal pruritus improved after delivery, and she was discharged on postpartum day 4. At 42 days postpartum, ALT was 67 U/L, AST 119 U/L, TBA 17.4 μmol/L, and total bilirubin 23.3 μmol/L. Magnetic resonance cholangiopancreatography (MRCP) was recommended to evaluate the biliary tree, but it had not been completed when the manuscript was revised. The reason for non-completion was not documented in the available record. All available serial biochemical parameters and treatment milestones are presented in Table 1, and their temporal relationships are illustrated in Figure 1.

**TABLE 1:** Serial maternal liver biochemical parameters and treatment milestones.

Gestational age	ALT (U/L)	AST (U/L)	TBA (μmol/L)	TBil (μmol/L)	Treatment or clinical milestone
Before referral (15 weeks)	382	791	53.8	—	UDCA and ademetionine started
15 + 3	909	465	224.6	12.9	Hospital admission; glutathione administered; initial multidisciplinary review
16 + 3	967	468	151.4	24.7	—
17 + 3	724	337	141.5	24.5	—
18 + 4	843	486	214.4	28.5	—
19 + 3	865	507	166.4	28.9	—
20 + 2	1,314	702	187.0	40.0	Polyene phosphatidylcholine added at 20 weeks
21 + 1	1,185	651	191.2	33.7	—
22 + 4	1,492	888	159.2	36.7	—
23 + 3	1,395	863	129.3	36.4	—
24 + 2	1,241	725	168.7	37.5	—
25 + 3	1,274	766	141.1	43.5	—
26 + 3	1,277	779	111.2	43.2	—
27 + 1	1,121	667	133.1	39.3	—
28 + 4	1,038	623	201.6	40.2	Last assessment before bicyclol
29 + 2	464	668	122.2	43.6	First assessment after bicyclol initiation at 29 weeks
30 + 3	218	595	145.2	44.7	—
31 + 4	186	505	96.7	46.5	—
32 + 6	158	532	146.6	42.8	—
34 + 0	137	278	139.5	45.9	Final antenatal assessment; repeat multidisciplinary consultation; cesarean delivery at 34+1 weeks
Postpartum day 42	67	119	17.4	23.3	Postpartum follow-up

Abbreviations: ALT, alanine aminotransferase; AST, aspartate aminotransferase; TBA, total bile acids; TBil, total bilirubin; UDCA, ursodeoxycholic acid.

The table includes all serial biochemical parameters documented in the medical record. Assessments were performed at approximately weekly intervals. An em dash indicates that no result was available for that time point. Concomitant treatments were continued unless otherwise specified. Bicyclol was initiated at 29 weeks of gestation at a dose of 50 mg three times daily and continued until cesarean delivery at 34+1 weeks.

## 3 Discussion

### 3.1 Diagnostic interpretation and differential diagnosis

The presentation supports a pregnancy-related cholestatic component, including palmoplantar pruritus, markedly elevated TBA, a similar presentation in a previous pregnancy, and postpartum improvement. However, onset at 15 weeks, previous choledocholithiasis and biliary surgery, progressive hyperbilirubinemia, a 10-mm common bile duct, and persistent abnormalities 42 days postpartum are atypical for classical ICP. The working diagnosis was therefore pregnancy-associated cholestasis with a possible ICP component superimposed on suspected chronic hepatobiliary dysfunction.

Possible structural causes include residual or recurrent choledocholithiasis, postoperative stricture, and occult biliary obstruction. A common bile duct diameter of up to 10 mm may reflect compensatory, non-obstructive dilatation after cholecystectomy (Park et al., 2012). This finding alone does not establish biliary obstruction. Postoperative anatomy could explain the dilatation but is unlikely to explain the overall presentation. However, progressive hyperbilirubinemia and persistent liver test abnormalities 42 days postpartum mean that a clinically relevant structural process cannot be excluded. Without MRCP, benign postoperative dilatation could not be reliably distinguished from structural biliary disease. This uncertainty also limits attribution of later biochemical changes to bicyclol.

An inherited bile transporter disorder is another possible explanation. Variants in *ABCB4, ABCB11, ATP8B1, TJP2*, and other cholestasis-related genes have been associated with adult-onset or pregnancy-associated liver disease (Nayagam et al., 2022; European Association for the Study of the Liver, 2024). In the absence of a cholestasis gene panel, MDR3- or BSEP-related disease cannot be excluded. Negative antinuclear, anti-smooth muscle, and anti-liver kidney microsomal antibody results do not exclude all autoimmune cholangiopathies. Antimitochondrial antibody and serum immunoglobulin results were unavailable. Bile acid fraction analysis, liver histology, and complete structural evaluation were also unavailable. These missing data represent important limitations and should be considered when interpreting this case.

### 3.2 Guideline context and selection of adjunctive treatment

Current SMFM, RCOG, and EASL guidelines recommend UDCA to improve maternal symptoms. Delivery planning is based primarily on bile acid concentration, gestational age, maternal symptoms, and coexisting disease (Society for Maternal-Fetal Medicine et al., 2021; Girling et al., 2022; European Association for the Study of the Liver, 2023). However, these guidelines do not recommend bicyclol and provide little guidance for very early cholestasis with atypical diagnostic features and persistent biochemical abnormalities despite combination therapy. In this patient, the UDCA dose of 1,000 mg/day corresponded to approximately 14.9 mg/kg/day, which is within the commonly recommended dose range.

The team also considered several alternative management strategies. Rifampicin has been used as adjunctive therapy for refractory ICP, but supporting evidence remains limited. In this patient, concerns about hepatotoxicity, drug interactions, and vitamin K deficiency made its use less favorable because of the marked liver biochemical abnormalities. Dexamethasone is not recommended as ICP treatment. Antenatal corticosteroids serve a different purpose and are indicated when preterm delivery is anticipated. Cholestyramine may further impair the absorption of fat-soluble vitamins. Plasma exchange and extracorporeal liver support are invasive interventions that are generally reserved for rescue therapy. Delivery at 29 weeks would also have exposed the fetus to substantial risks associated with extreme prematurity. Against this background, bicyclol was used as an off-label adjunct based on evidence from nonpregnant populations and multidisciplinary clinical judgment, with the intention of targeting hepatocellular injury. This rationale explains the clinical decision but should not be interpreted as evidence supporting the use of bicyclol in pregnancy (Walker et al., 2020; European Association for the Study of the Liver, 2023).

### 3.3 Pharmacological rationale and cautious interpretation of discordant biochemical trends

Bicyclol is a synthetic hepatoprotective agent with antioxidant, anti-inflammatory, mitochondrial-protective, and cell-death regulatory effects in experimental studies (Zhao et al., 2021; 2022; Liu et al., 2025). In a phase II trial in nonpregnant adults with drug-induced liver injury, it produced greater ALT reductions and faster normalization than polyene phosphatidylcholine (Tang et al., 2022). These data provide a rationale for its use in hepatocellular injury but do not establish efficacy in pregnancy-associated cholestasis, anticholestatic activity, or pregnancy safety.

At 29 weeks, bicyclol was added after documented ALT and AST peaks of 1,492 and 888 U/L, respectively, despite UDCA-based combination therapy. ALT remained above 1,000 U/L through 28+1 weeks, fell to 464 U/L at 29+2 weeks, and remained at 137–218 U/L from 30 + 1 to 34+0 weeks. AST also declined but remained abnormal throughout follow-up. The later decline was temporally associated with bicyclol exposure but does not establish a treatment effect. TBA showed no sustained decline (96.7–146.6 μmol/L), and total bilirubin remained elevated (42.8–46.5 μmol/L). This discordance argues against overall cholestatic improvement and may reflect a greater effect on hepatocellular injury than on cholestatic dysfunction. However, persistent structural or genetic hepatobiliary disease could equally explain the pattern. Grade III meconium-stained amniotic fluid showed that fetal risk persisted but could not be attributed to bicyclol.

Causal interpretation is limited by pretreatment fluctuations, multidrug therapy, advancing gestation, unmeasured adherence, and the absence of a control group or dechallenge–rechallenge sequence. These factors prevent isolation of an independent bicyclol effect, although a contribution could not be excluded. The case should therefore not be interpreted as evidence of improved clinical outcomes or prolonged pregnancy. It should be viewed as hypothesis-generating, and further study is warranted.

### 3.4 Safety considerations for bicyclol use in pregnancy

Evidence regarding bicyclol exposure during human pregnancy remains very limited. A rat study found no reproductive toxicity or fetal malformations after exposure during organogenesis (Liu et al., 2005), while a phase II trial reported short-term tolerability in nonpregnant adults (Tang et al., 2022). Neither finding establishes pregnancy safety. Data on placental transfer, pregnancy pharmacokinetics, dose selection, and long-term offspring outcomes are lacking.

In this patient, bicyclol was administered from 29 weeks of gestation until delivery at 34+1 weeks, representing approximately 5 weeks of exposure. No clinically apparent maternal adverse events were documented during treatment. The infant had normal Apgar scores and no clinically apparent early neonatal complications. These observations provide only limited information on short-term tolerability. They do not establish maternal or fetal safety and cannot exclude uncommon or delayed adverse effects. Interpretation is further limited by concomitant therapy, prematurity, severe maternal cholestasis, the absence of systematic adverse-event assessment and placental histopathological examination, and the lack of long-term follow-up of infant growth and neurodevelopment. Within these limitations, no obvious drug-related safety signal was identified during the observed period. However, this case should not be interpreted as evidence supporting the routine use of bicyclol during pregnancy. Any use during pregnancy should remain individualized risk-benefit assessment, informed consent, and close maternal and fetal monitoring until more robust evidence becomes available.

### 3.5 Clinical implications, postpartum considerations, and limitations

This case highlights the challenges of managing severe pregnancy-associated cholestasis in the setting of suspected underlying hepatobiliary dysfunction. After bicyclol was added, aminotransferase levels declined later in pregnancy, whereas TBA remained markedly elevated and hyperbilirubinemia persisted. This temporal association raises the possibility that bicyclol may have contributed to the observed aminotransferase decline. However, it does not establish a treatment effect or an anticholestatic effect. The case also could not establish that bicyclol prolonged pregnancy.

Persistent liver test abnormalities 42 days after delivery, together with disease onset at 15 weeks of gestation, previous common bile duct surgery, and mild extrahepatic bile duct dilatation, are atypical for classical ICP and raise suspicion of an underlying hepatobiliary disorder. Because liver biochemical abnormalities in classical ICP typically resolve after delivery, further postpartum evaluation is warranted (European Association for the Study of the Liver, 2023; Dajti et al., 2025). MRCP may help identify recurrent choledocholithiasis, postoperative biliary stricture, or other causes of biliary obstruction. If no structural abnormality is identified, genetic testing for cholestasis-associated genes, including *ABCB4, ABCB11, ATP8B1, and TJP2*, should be considered.

Strengths include the rare presentation, multidisciplinary care, and serial biochemical monitoring. However, this uncontrolled case lacked an interpretable dechallenge-rechallenge sequence and formal time-series analysis, precluding causal inference. Pruritus was not graded with a validated scale, adherence was not assessed systematically, and some scheduled laboratory results were unavailable. Thus, exposure to bicyclol and concomitant treatments was uncertain. No bile acid fractionation was performed, limiting interpretation of individual species and TBA trends. Pregnancy-specific bicyclol pharmacokinetics, maternal and cord blood concentrations, and placental transfer were not assessed. Lack of liver histology limited evaluation of chronic hepatocellular or cholangiopathic disease. Without placental pathology, placental injury and possible meconium-associated changes could not be evaluated. Neonatal follow-up was confined to the early postnatal period, without long-term assessment of growth, liver function, or neurodevelopment. Case-report publication bias may favor unusual or apparently favorable findings over neutral or unfavorable findings. These limitations preclude conclusions about efficacy, causality, optimal dosing, pregnancy duration, or maternal and fetal safety.

## 4 Conclusion

This case reports severe early-onset pregnancy-associated cholestasis in a woman with suspected underlying hepatobiliary dysfunction. During adjunctive bicyclol exposure, aminotransferase levels declined later in pregnancy, whereas TBA remained markedly elevated and hyperbilirubinemia persisted. This discordant biochemical pattern may reflect differential changes in hepatocellular injury and cholestatic dysfunction. However, this observation could not support causal inference from a single case. Pretreatment biochemical variability and continued multidrug therapy prevent the observed changes from being attributed independently to bicyclol. No clinically apparent maternal adverse events were documented, and no clinically apparent early neonatal complications were observed. However, evidence from a single mother-infant pair is insufficient to establish the safety of bicyclol during pregnancy. Persistent postpartum biochemical abnormalities support further evaluation for underlying structural and genetic hepatobiliary disease. This case should therefore be regarded as hypothesis-generating rather than as evidence of efficacy, safety, or an effect on pregnancy duration. Further prospective studies with comprehensive diagnostic evaluation and long-term maternal and infant follow-up are needed to determine whether bicyclol has a role in the management of pregnancy-associated cholestasis.

## Data Availability

The raw data supporting the conclusions of this article will be made available by the authors, without undue reservation.
